# Diagnostic accuracy of plasma p‐tau217/Aβ42 for Alzheimer's disease in clinical and community cohorts

**DOI:** 10.1002/alz.70038

**Published:** 2025-03-29

**Authors:** Jun Wang, Shan Huang, Guoyu Lan, Yu‐Jie Lai, Qing‐Hua Wang, Yang Chen, Zhong‐Song Xiao, Xiao Chen, Xian‐Le Bu, Yu‐Hui Liu, Fan Zeng, Laihong Zhang, Anqi Li, Yue Cai, Pan Sun, Zhengbo He, Vincent Doré, Jurgen Fripp, Pierrick Bourgeat, Qin Chen, Jin‐Tai Yu, Yi Tang, Henrik Zetterberg, Colin L. Masters, Tengfei Guo, Yan‐Jiang Wang, for the Translational Biomarker Research of AgIng and Neurodegeneration (TBRAIN)

**Affiliations:** ^1^ Department of Neurology and Center for Clinical Neuroscience Daping Hospital, Third Military Medical University Chongqing China; ^2^ Chongqing Key Laboratory of Ageing and Brain Diseases Chongqing China; ^3^ Institute of Biomedical Engineering Shenzhen Bay Laboratory Shenzhen China; ^4^ Chongqing Institute for Brain and Intelligence Guangyang Bay Laboratory Chongqing China; ^5^ Department of Nuclear Medicine Daping Hospital, Third Military Medical University Chongqing China; ^6^ Australian eHealth Research Centre CSIRO Health and Biosecurity Brisbane Queensland Australia; ^7^ Department of Molecular Imaging & Therapy Austin Health Melbourne Victoria Australia; ^8^ Department of Neurology West China Hospital of Sichuan University Chengdu China; ^9^ Department of Neurology and National Center for Neurological Diseases, Huashan Hospital, State Key Laboratory of Medical Neurobiology and MOE Frontiers Center for Brain Science, Shanghai Medical College Fudan University Shanghai China; ^10^ Department of Neurology & Innovation Center for Neurological Disorders Xuanwu Hospital, Capital Medical University, National Center for Neurological Disorders Beijing China; ^11^ Department of Psychiatry and Neurochemistry Institute of Neuroscience and Physiology The Sahlgrenska Academy at the University of Gothenburg Mölndal Sweden; ^12^ Clinical Neurochemistry Laboratory Sahlgrenska University Hospital Mölndal Sweden; ^13^ Department of Neurodegenerative Disease UCL Institute of Neurology, Queen Square London UK; ^14^ UK Dementia Research Institute at UCL London UK; ^15^ Hong Kong Center for Neurodegenerative Diseases Clear Water Bay Hong Kong China; ^16^ Wisconsin Alzheimer's Disease Research Center University of Wisconsin School of Medicine and Public Health, University of Wisconsin‐Madison Madison Wisconsin USA; ^17^ The Florey Institute of Neuroscience and Mental Health University of Melbourne, Parkville Melbourne Victoria Australia

**Keywords:** Alzheimer's disease, amyloid positron emission tomography, blood biomarker, diagnosis, Lumipulse, p‐tau217/Aβ42, two‐cutoff approach

## Abstract

**INTRODUCTION:**

This study was undertaken to evaluate the diagnostic performance of a novel plasma phosphorylated tau (p‐tau) 217/amyloid beta (Aβ) 42 ratio test for Alzheimer's disease (AD).

**METHODS:**

The diagnostic performance of the Lumipulse G plasma p‐tau217/Aβ42 ratio was evaluated using Aβ and tau positron emission tomography (PET) as reference standards in a clinic cohort (*n* = 391) and a community cohort (*n* = 121).

**RESULTS:**

Plasma p‐tau217/Aβ42 exhibited high performance for abnormal statuses of Aβ PET (area under the curve [AUC]: 0.963 to 0.966) and tau PET (AUC: 0.947 to 0.974), which were clinically equivalent to those of cerebrospinal fluid (CSF) p‐tau181/Aβ42 and Aβ42/Aβ40 and higher than those of blood p‐tau217, Aβ42/Aβ40, p‐tau181, and p‐tau181/Aβ42 in both clinic and community cohorts. Applying a two‐cutoff approach improved the specificity without reducing sensitivity. The p‐tau217/Aβ42 ratio had a lower intermediate percentage than p‐tau217 alone in both clinic (10.6% vs 13.0%) and community (16.5% vs 31.4%) cohorts.

**DISCUSSION:**

Plasma p‐tau217/Aβ42 has high performance in detecting cerebral AD pathologies, thus offering a promising tool for clinical diagnosis and community screening of AD.

**Highlights:**

- Lumipulse G plasma p‐tau217 and the p‐tau217/Aβ42 ratio accurately identified abnormal Aβ and tau PET statuses in both clinical and community cohorts.
- The performance of plasma p‐tau217 and p‐tau217/Aβ42 ratio were equivalent to CSF tests.
- Plasma p‐tau217/Aβ42 ratio outperformed p‐tau217 alone in identifying Aβ PET positivity, and this superiority is more obvious in the community cohort, suggesting an advantage in the early diagnosis of AD.
- Two cut points of p‐tau217/Aβ42 were established in the Chinese population for clinical laboratory and community screening uses.

## 1 BACKGROUND

Approximately 55.2 million people worldwide are living with dementia, and this figure is predicted to increase to 78 million by 2030 and to 139 million by 2050.^1^ Addressing dementia has been proposed as one of the greatest health challenges by the World Health Organization (WHO), and improving diagnosis has been specified as a key area. Therefore, developing highly sensitive, cost‐effective, disease‐specific diagnostic biomarkers is a strategic goal. Alzheimer's disease (AD) accounts for 60% to 70% of all dementia cases. The rate of AD misdiagnosis is approximately 20% to 35% in specialty clinics and higher than 50% in primary care clinics without biomarkers.^2^, ^3^, ^4^ Early, accurate, and biomarker‐based diagnosis of AD is imperative, especially as disease‐modifying treatments (eg, lecanemab and donanemab) become available.^5^, ^6^, ^7^

Positron emission tomography (PET) and cerebrospinal fluid (CSF) biomarkers have been incorporated into the diagnostic framework for AD with high diagnostic accuracy.^8^, ^9^, ^10^, ^11^ However, their widespread clinical use has been limited by their high costs, insufficient availability, and invasive nature. Blood biomarkers have emerged and surged in recent years as scalable and cost‐effective tools that can substantially reduce the reliance on CSF or PET scans, thereby expanding their application and reducing medical and economic burdens.^12^, ^13^, ^14^ The plasma amyloid beta (Aβ) 42/40 ratio and phosphorylated tau (p‐tau) are disease‐specific biomarkers of Aβ and tau pathologies and have been recommended in recent Alzheimer's Association criteria for diagnosing AD when shown to have at least 90% accuracy in comparison to amyloid PET or CSF assays.^15^ The diagnostic performance of plasma Aβ42/40 is relatively lower with the area under the receiver operating characteristic (ROC) curve (AUC) of less than 90%,^16^, ^17^, ^18^ while p‐tau (eg, tau phosphorylated at threonine 217 [p‐tau217], p‐tau231, p‐tau181) have been shown to perform better at detecting AD pathology and differentiating AD from other neurodegenerative disorders.^13^, ^19^, ^20^, ^21^ Recently, plasma p‐tau217 analyzed by mass spectrometry and ALZpath Simoa Assay exhibited the highest performance, with an AUC of 0.92 to 0.97 for identifying Aβ PET positivity; these methods were even clinically equivalent or superior to the US FDA‐approved CSF tests.^22^, ^23^

The new test, Lumipulse G p‐tau217/Aβ42 plasma ratio, has received Breakthrough Device Designation from the FDA and been filed with the FDA as a commercially available blood‐based in vitro diagnostic (IVD) test of AD. The Lumipulse plasma p‐tau217 individually exhibited diagnostic performance as accurately as ALZpath SIMOA p‐tau217 in identifying CSF Aβ status (AUC = 0.92 to 0.95) in very recent studies.^24^, ^25^, ^26^, ^27^, ^28^ The new ratio test complements the Lumipulse G CSF Aβ42/40 ratio, authorized by the FDA in 2022. We initiated the Translational Biomarker Research of AgIng and Neurodegeneration (TBRAIN) consortium in China, intending to translate biomarkers of aging and age‐related neurodegenerative diseases (eg, AD) from research into clinical practice. In this study, we investigated the diagnostic accuracy of Lumipulse G plasma p‐tau217/Aβ42, in comparison with other blood biomarkers and FDA‐approved Lumipulse G CSF Aβ42/40, in identifying abnormal Aβ and tau PET statuses in two settings: (1) in patients with cognitive symptoms or concerns in a real‐world clinical cohort and (2) in community‐based populations willing to perform PET scans for identifying cerebral amyloid and tau accumulation. We also defined the corresponding cutoffs in the two settings in the Chinese population.

## 2 METHODS

### 2.1 Study participants

This study included participants from one memory clinic cohort and one community‐based cohort. All participants provided written informed consent, and the studies were approved by the relevant ethics boards. This study followed the Strengthening the Reporting of Observational Studies in Epidemiology (STROBE) reporting guidelines.

**RESEARCH IN CONTEXT**

1. **Systematic review**: The authors reviewed literature in PubMed related to plasma p‐tau217 and p‐tau217/Aβ42 ratio for identifying brain amyloid pathology related to AD. A limited number of publications on Lumipulse plasma p‐tau217/Aβ42 ratio tests were identified. These publications are properly cited throughout the article.
2. **Interpretation**: Both Lumipulse G plasma p‐tau217 alone and the p‐tau217/Aβ42 ratio showed high diagnostic accuracy for identifying brain amyloid deposition in both clinical and community cohorts, which were equivalent to FDA‐approved Lumipulse CSF tests. Applying the two‐cutoff approach improved the overall accuracy and specificity without reducing sensitivity, and the plasma p‐tau217/Aβ42 ratio had a lower intermediate percentage than p‐tau217 alone.
3. **Future directions**: Further studies need to validate the cutoffs in prospective and independent cohorts for implementation in real‐world clinical and community settings.

#### 2.1.1 The clinical cohort: Chongqing Ageing & Dementia Study (CADS) cohort

The CADS is a real‐world, longitudinal cohort study that began recruiting patients with complaints of cognitive decline or consulting for the risk of developing AD from the tertiary memory clinic at Daping Hospital in Chongqing in 2010. This study was approved by the Institutional Review Board of Daping Hospital, and all participants and their caregivers provided written informed consent (Chinese Clinical Trial Registry No.: ChiCTR1900027622).

The present study included all individuals in the CADS cohort who had plasma samples for assays and underwent Aβ and/or tau PET scans within 1 year of blood collection between January 2015 and May 2024. The included participants were classified as cognitively unimpaired (CU) control, subjective cognitive decline (SCD), mild cognitive impairment (MCI), and dementia due to AD or other causes. CU individuals had no cognitive concerns and a Clinical Dementia Rating (CDR) score of 0. SCD individuals had complaints of cognitive decline but no objective cognitive impairment, with a CDR score of 0.5. Individuals with MCI had subjective and objective cognitive impairment and a CDR score of 0.5 and did not meet the Diagnostic and Statistical Manual of Mental Disorders, Fifth Edition, criteria for dementia. Individuals with dementia had a CDR score of 1 to 3.

#### 2.1.2 The community cohort: The Greater‐Bay‐Area Healthy Aging Brain Study (GHABS) cohort

The GHABS is a multicenter and prospective population‐based study initiated in 2021 in the Guangdong‐Hong Kong‐Macao Greater‐Bay‐Area of South China. Detailed information is given elsewhere.^4^ The GHABS was approved by the Shenzhen Bay Laboratory's and the collaborating hospitals’ ethics committees. All participants signed written informed consent forms. Participants (*n* = 121) who had both Aβ and tau PET scans were included in this study, including 72 CU individuals, 23 with MCI, and 26 with dementia. The participants were classified as CN, MCI, or AD dementia, following the standard protocol of the ADNI cohort. CU participants were normal in the Mini‐Mental State Examination (MMSE), logical memory recall (LMR), and activities of daily living (ADL), and their CDR score was 0. Participants with MCI had impaired LMR and a CDR score of 0.5, with a mandatory requirement of the memory box score being 0.5 or greater, but had normal MMSE and ADL scores. Dementia due to AD was abnormal in MMSE, logical memory, and ADL, as well as a CDR score of 0.5 or greater.

### 2.2 Image acquisition and processing

In CADS, Aβ PET is performed with [^11^C]Pittsburgh compound B (PiB) or [^18^F]florbetapir (AV45) and tau PET with [^18^F]MK‐6240 tracers. Both Aβ and tau PET images were analyzed using Computational Analysis of PET by the Australian Imaging, Biomarker and Lifestyle Flagship Study (CapAIBL), a cloud‐based platform in which PET images are spatially normalized to a standard template via an adaptive atlas approach (https://capaibl‐milxcloud.csiro.au). The Aβ PET scans were quantified using standard Centiloids (CLs), and CL > 25 or > 15 was selected to define a high Aβ (positive) scan.^29^ The tau PET scans were quantified using the CenTauR z score (CTR_z_), a universal standard scale across tracers similar to that used in the Centiloid project and generated from the mean and standard deviation (SD) of standardized uptake value ratio (SUVR) in the cognitively unimpaired Aβ‐negative subjects. A threshold of >2 CTR_z_ in the meta‐temporal region of interest (ROI) or the mesial temporal region was used to define a positive tau scan.^30^ Meanwhile, Aβ and tau PET were also visually read in a grayscale and rainbow color scale using a dichotomous negative/positive by two experienced PET diagnosticians blinded to clinical information and quantitative results. Aβ PET imaging was read according to a FDA‐approved protocol and was classified positive when cortical activity was equal to or greater than white matter activity in one or more lobes or the cerebellar white matter. Tau PET was visually analyzed according to the visual read algorithm used by Seibyl et al. for assessing [^18^F]MK‐6240 PET scans.^31^

In GHABS, Aβ PET was performed with [^18^F]‐AV45 or [^18^F]N‐methylderivative of Amyvid (D3FSP) tracers.^32^ The D3FSP and AV45 SUVR of AD summary cortical regions (posterior cingulate cortex, precuneus, frontal lobe, parietal lobe, and lateral temporal) were obtained by dividing the radiotracer uptake value of AD typical brain regions by that in the whole cerebellum. The thresholds of Aβ PET positivity were defined as D3FSP COMPOSITE SUVR ≥0.78^32^ and AV45 SUVR ≥ 1.11.^33^ Tau PET was performed with [^18^F]‐flortaucipir (AV1451). The SUVR of the AD temporal‐meta‐ROI (entorhinal cortex, parahippocampal gyrus, fusiform, amygdala, inferior temporal, and middle temporal brain regions) was used to evaluate cortical tau deposition, taking the inferior cerebellar cortex as the reference brain region, and a threshold of ≥1.27 was used to define a positive tau PET. ^4^

There was high agreement between quantitative and visual reads for Aβ PET status in our cohorts, with 94.3% in CADS and 94.2% in GHABS (Table S1).

### 2.3 Diagnosis of AD

The diagnosis of AD in two cohorts was according to the 2024 Alzheimer's Association criteria,^15^ and subjects were classified into preclinical, prodromal, and AD dementia. When identifying AD versus non‐AD in the CADS cohort in the present study, the clinical‐biological construct recommended by the international working group was adopted^34^: Patients who were clinically eligible for probable/possible AD according to the National Institute of Neurological and Communicative Disorders and the Stroke‐Alzheimer's Disease and Related Disorders Association (NINCDS‐ADRDA) criteria or amnestic MCI according to the Petersen et al. criteria^35^ and had positive Aβ PET results were diagnosed with AD. Those who were clinically diagnosed with other neurodegenerative diseases (such as dementia with Lewy bodies [DLB], multiple system atrophy) but who were positive for Aβ and/or tau PET were considered to have comorbid AD pathology and were put into the non‐AD group. Biological AD was determined by both Aβ and tau PET positivity regardless of the clinical diagnosis.^11^

### 2.4 CSF and plasma biomarkers

CSF samples were collected via lumbar puncture under local anesthesia and were centrifuged at 2000 × *g* for 10 min at room temperature, aliquoted, and stored at −80°C within 2 h after collection. CSF Aβ42, Aβ40, p‐tau181, and t‐tau concentrations were measured on the fully automated Lumipulse G1200 platform via commercially available kits (Fujirebio Europe, Ghent, Belgium). The ratio of CSF Aβ42 to Aβ40 (Aβ42/40) was measured by Lumipulse assays, which have received FDA approval for detecting cerebral amyloid deposition associated with AD in individuals with cognitive impairment.

For CADS, fasting blood was collected in EDTA‐containing tubes and centrifuged at 2000 × *g* for 10 min at room temperature to separate the plasma. The samples were aliquoted and stored at −80°C within 2 h of blood collection. For GHABS, after collection, the fasting blood was placed in an incubator at 4°C and shipped back to the laboratory within 30 min for subsequent analysis. The blood was centrifuged at 1600 × *g* for 15 min at 4°C. The upper plasma layer was separated and centrifuged again at 16,000 × *g* for 15 min at 4°C, after which the supernatant was aliquoted and stored at −80°C until use. Plasma Aβ42, Aβ40, p‐tau181, and p‐tau217 concentrations were quantified on the Lumipulse G1200 platform.

Both CSF and plasma samples from the CADS and GHABS cohorts were analyzed in the laboratory at Daping Hospital, which is a member of the Alzheimer's Association Quality Control Program for CSF and blood biomarkers. The within‐assay run variability and within‐laboratory longitudinal variability of quality controls for CSF and plasma tests are reported in Table S2.

### 2.5 Statistical analysis

The performance of plasma and CSF biomarkers in identifying Aβ and tau PET statuses and AD diagnosis was evaluated using the ROC curves. AUCs with 95% CI were calculated with the pROC package. The DeLong test was used to compare the mean differences with 95% CIs between plasma p‐tau217 and other plasma biomarkers in the entire cohort, and between CSF and plasma biomarkers in the CSF subset. The single‐cutoff approach derives a binary reference (positive/negative) based on the max Youden index using the cutpoint package. The two‐cutoff approach derives three‐range references (positive/uncertain/negative). The lower threshold was obtained by maximizing the specificity with the sensitivity fixed at 95%, whereas the upper threshold was obtained by maximizing the sensitivity with the specificity fixed at 95%. Participants with biomarker levels between these two thresholds were categorized as intermediate.

Statistics were calculated as the mean with 95% CI of the bootstrapped sample (*n* = 1000 resamples with replacement stratifying by the output), including the difference in all other biomarkers from plasma p‐tau217 or CSF biomarkers (reference). We considered the compared biomarkers clinically equivalent if the 95% CI of the mean difference included zero.

All analyses were performed using R version 4.2.2 (R Project for Statistical Computing), with a two‐sided *α* of 0.05. Figures were generated using Graphpad Prism version 10.0.

## 3 RESULTS

### 3.1 Study participants

A total of 512 participants were included in this study (Table 1). The clinical cohort (CADS cohort) included 391 participants with a mean (SD) age of 66.22 (9.75) years, of whom 214 (54.73%) were female. In this cohort, 44 (11.25%) individuals had normal cognition or subjective cognitive decline (SCD), 146 (37.34%) had MCI, and 201 (51.41%) had dementia. Table S3 in the supporting information describes the diagnosis of patients with impaired cognition. The community‐based cohort (GHABS cohort) included 121 individuals, with a mean age of 66.61 (8.73) years, of whom 75 (61.98%) were female, and 70 (57.85%) were cognitively intact.

**TABLE 1:** Participant characteristics.

	CADS				GHABS			
Characteristic	All (*n* = 391)	CU (*n* = 44)	MCI (*n* = 146)	Dementia (*n* = 201)	All (*n* = 121)	CU (*n* = 72)	MCI (*n* = 23)	Dementia (*n* = 26)
Age, years	66.22 (9.75)	61.07 (9.97)	66.34 (9.54)	67.26 (9.54)	66.61 (8.73)	65.34 (8.27)	69.93 (8.03)	67.17 (9.97)
Female, *n* (%)	214 (54.73)	29 (65.91)	75 (51.37)	110 (54.73)	75 (61.98)	44 (62.86)	14 (60.87)	17 (65.38)
Education, years^a^	9.89 (3.91)	11.65 (3.66)	10.45 (3.73)	9.10 (3.92)	12.37 (3.98)	13.83 (3.24)	10.98 (4.31)	9.54 (3.67)
APOE ε4 carriers, No. (%)^b^	136 (34.96)	6 (13.95)	50 (34.48)	80 (39.80)	54 (44.63)	29 (40.28)	9 (39.13)	16 (61.54)
MMSE score^c^	19.43 (7.50)	28.05 (1.73)	24.61 (2.96)	13.88 (5.97)	25.08 (5.84)	28.37 (1.72)	25.17 (2.71)	15.25 (4.85)
Aβ PET (positive No./total No., %)	184/385 (47.79)	0/43 (0)	48/141 (34.04)	136/201 (67.66)	54/121 (44.63)	18/72 (25.00)	13/23 (56.52)	23/26 (88.46)
Tau PET (positive No./total No., %)	81/196 (41.33)	0/20 (0)	20/76 (26.32)	64/100 (64.00)	44/121 (36.36)	7/72 (9.72)	14/23 (60.87)	23/26 (88.46)

*Notes*: All the data are represented as mean (SD) unless otherwise stated. Percentages are calculated from the sample available for each variable. Aβ and Tau PET positivity were based on visual read.

Abbreviations: CU, cognitively unimpaired; MCI, mild cognitive impairment; MMSE, Mini‐Mental State Examination; CADS, Chongqing Ageing & Dementia Study; GHABS, Greater‐Bay‐Area Healthy Aging Brain Study.

^a^ Nine participants missing in CADS.

^b^ Two participants missing in CADS.

^c^ Five participants missing in CADS and three in GHABS.

### 3.2 Associations of plasma biomarkers with brain biomarkers

**FIGURE 1:**
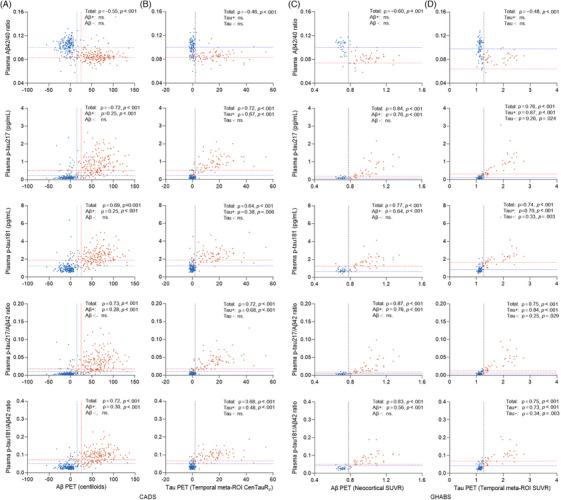
Correlations of plasma biomarkers with Aβ and tau PET. (A and B) Correlations of plasma biomarkers with brain Aβ burden quantified by Centiloids (A) and tau burden quantified by temporal meta‐ROI CenTauR_z_ (B) in CADS cohort. (C and D) Correlations of plasma biomarkers with brain Aβ burden quantified by neocortical SUVR of D3FSP tracer (C) and tau burden by temporal meta‐ROI SUVR of AV1451 tracer (D) in the GHABS cohort. Aβ, amyloid beta; CADS, Chongqing Ageing & Dementia Study; GHABS, Greater‐Bay‐Area Healthy Aging Brain Study; PET, positron emission tomography; ROI, region of interest; SUVR, standardized uptake value ratio.

All the CSF and plasma samples were quantifiable with biomarker concentrations within the measurement range (Table S2). We first investigated the associations of plasma biomarkers with the brain burdens of Aβ and tau as measured by PET. In the CADS cohort, plasma Aβ42/40 levels were negatively correlated with both Aβ Centiloids and temporal meta‐ROI CTR_z_ in the entire cohort, but not in the Aβ PET(+), Aβ PET(−), tau PET(+), or tau PET(−) subgroups. In contrast, the plasma p‐tau217 and p‐tau181 levels and their ratios to Aβ42 were positively correlated with Aβ and tau burdens in both the total sample and the Aβ PET(+) and tau PET(+) subgroups (Figure 1A,B). Similar results were observed in the GHABS cohort (Figure 1C,D). In the head‐to‐head comparisons, the correlations of p‐tau217 and p‐tau217/Aβ42 with the tau PET temporal meta‐ROI SUVR were much stronger than those with Aβ deposition in both the Aβ PET(+) and tau PET(+) subgroups (Table S4).

We also evaluated the associations between plasma and CSF biomarkers in the CSF subset of the CADS cohort (*n* = 150). In the total sample, plasma Aβ42/40 was positively associated with CSF Aβ42/40, whereas plasma p‐tau217, p‐tau181, and their ratios to Aβ42 were strongly associated with CSF p‐tau181 and p‐tau181/Aβ42. Similarly, except for the plasma Aβ42/40 ratio, plasma p‐tau217, p‐tau181, and the p‐tau217/Aβ42 and p‐tau181/Aβ42 ratios were positively associated with CSF p‐tau181 and p‐tau181/Aβ42 in the Aβ PET(+) subgroups (Figure S1).

### 3.3 Performance of blood biomarkers in classification of Aβ and tau PET status

**FIGURE 2:**
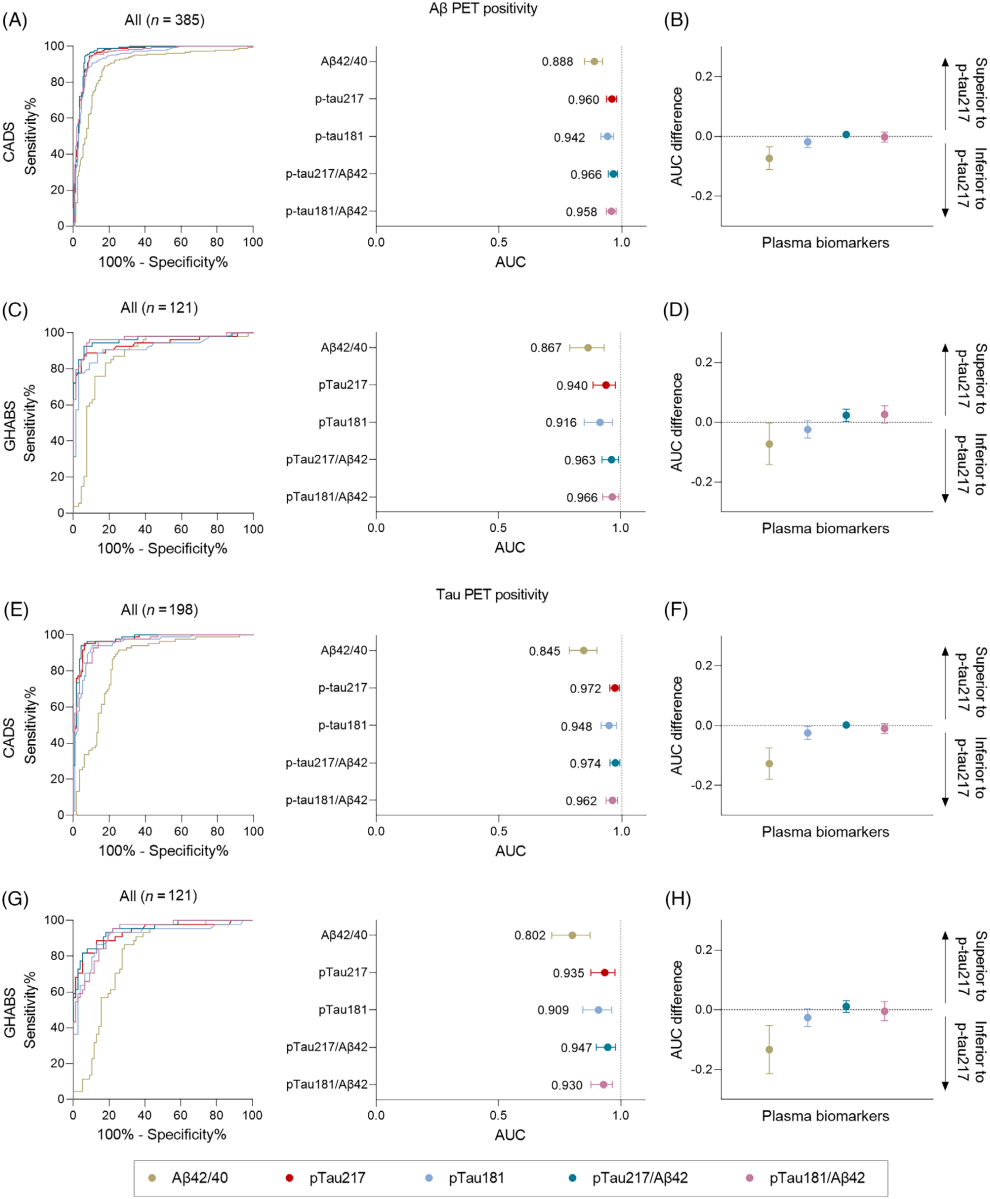
Performance of plasma biomarkers in classification of Aβ and tau PET statuses by visual read. (A, C, E, G) ROC curve and AUC of plasma biomarkers in classification of Aβ (A and C) and tau PET (E and G) status in the entire CADS (A and E) and GHABS (C and G) cohorts. Vertical dashed lines: AUC = 1. (B, D, F, H) Bootstrapped differences (*n* = 1000 resamples with replacement stratifying by output) between statistics using plasma p‐tau217 (reference) and other plasma biomarkers in CADS (B and F) and GHABS (D and H). Horizontal dashed line plotted at zero: lack of difference between plasma p‐tau217 and other plasma biomarkers. Other plasma biomarkers were considered to be clinically equivalent to p‐tau217 if the 95% CI of the mean difference included zero and clinically superior (>0) or inferior (<0) if it did not include zero. The dots and error bars represent the actual statistics and 95% CIs (from bootstrapped *n* = 1000 samples with replacement), respectively. AUC, area under the curve; CADS, Chongqing Ageing & Dementia Study; CI, confidence interval; GHABS, Greater‐Bay‐Area Healthy Aging Brain Study; PET, positron emission tomography; p‐tau, phosphorylated tau; ROC, receiver operating characteristic.

**FIGURE 3:**
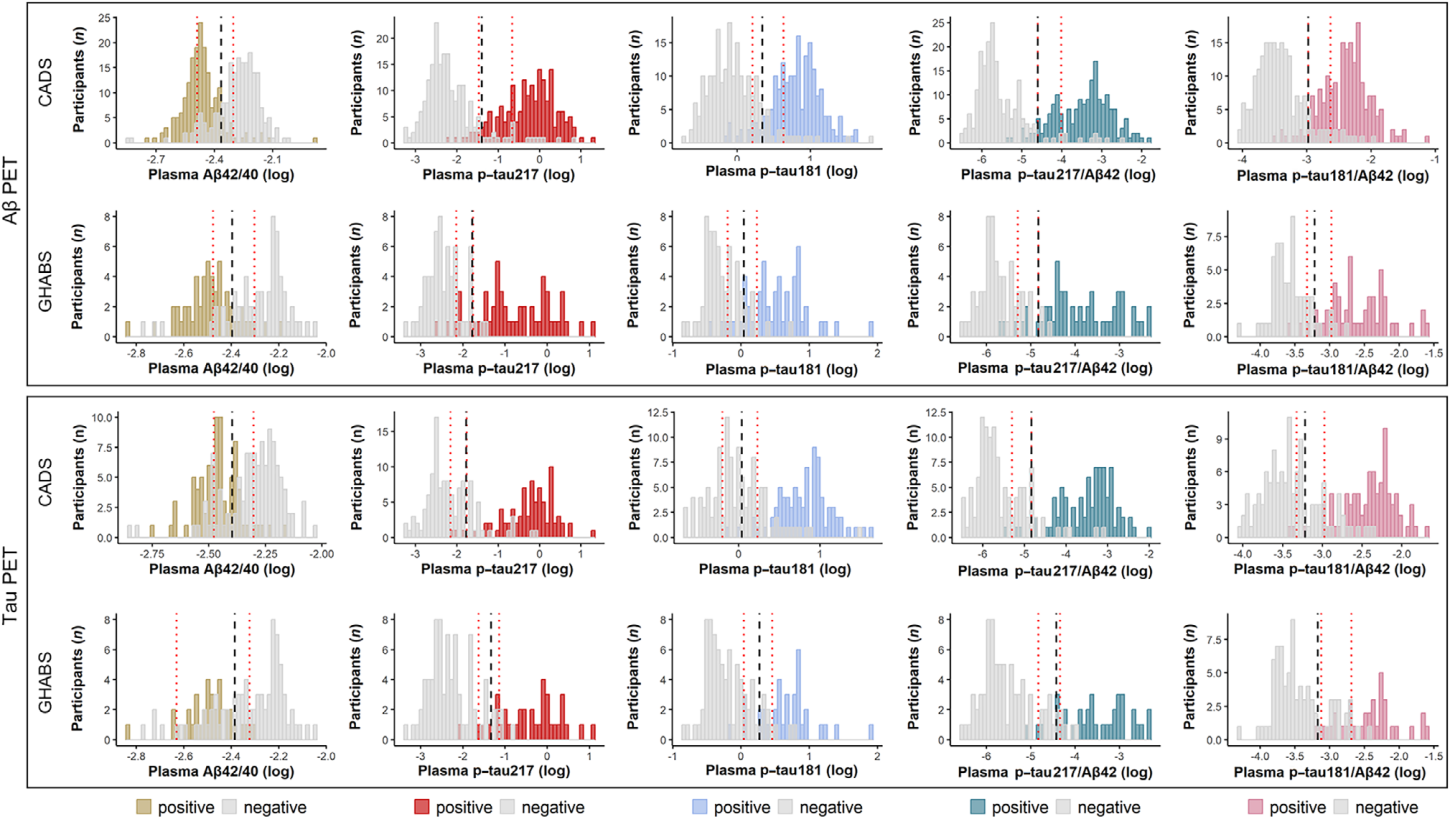
Frequency distribution of plasma biomarkers in CADS and GHABS cohorts. Histograms represent distribution of data colored by Aβ and tau PET imaging status. Vertical black line: threshold derived from single‐cutoff approach; red lines: lower and upper thresholds from two‐cutoff approach. Aβ and tau PET positivity were assessed by visual read method. Aβ, amyloid beta; PET, positron emission tomography.

We evaluated the performance of plasma biomarkers in the classification of Aβ PET or tau PET status. In the classification of Aβ PET status (visual read) in the entire CADS cohort, the plasma p‐tau217 level exhibited high performance (AUC = 0.960, 95% CI: 0.938, 0.979), and the p‐tau217/Aβ42 ratio slightly improved the AUC to 0.966 (95% CI: 0.945, 0.983; *p* < .05). Plasma p‐tau181 and p‐tau181/Aβ42 showed a slightly lower AUC but were not statistically significant (p‐tau181: AUC = 0.942, 95% CI: 0.915, 0.966; p‐tau181/Aβ42: AUC = 0.958, 95% CI: 0.937, 0.977; *p* > .05). Plasma Aβ42/40 exhibited good performance, although it was inferior to p‐tau (AUC = 0.888, 95% CI: 0.848, 0.922; *p* < .05) (Figure 2A,B and eTable 1). The distribution of blood biomarker levels was in good agreement with the classification of PET status, showing a bimodal pattern (Figure 3). In the sensitivity analysis, plasma p‐tau217 and p‐tau217/Aβ42 also performed best when Aβ PET positivity was defined as Centiloids > 25 (p‐tau217: AUC = 0.927, 95% CI: 0.897, 0.953; p‐tau217/Aβ42: AUC = 0.933, 95% CI: 0.902, 0.957) or > 15 (p‐tau217: AUC = 0.929, 95% CI: 0.901, 0.956; p‐tau217/Aβ42: AUC = 0.934, 95% CI: 0.906, 0.960) in the CADS cohort (Figure S2 and eTable 2). Similar results were obtained in the GHABS cohort: The AUC for plasma p‐tau217 (0.940, 95% CI: 0.887, 0.979) was similar to that of p‐tau181 (p‐tau181: AUC = 0.916, 95% CI: 0.851, 0.966; *p* > .05) and was superior to that of Aβ40/42 (Aβ40/42: AUC = 0.867, 95% CI: 0.792, 0.932; *p* < .05), whereas the p‐tau217/Aβ42 ratio improved the diagnostic performance (AUC = 0.963, 95% CI: 0.924, 0.992; *p* < .05) (Figure 2C,D and eTable 1). When using quantitative assessment of Aβ PET, plasma p‐tau217 and p‐tau217/Aβ42 also exhibited higher performance (p‐tau217: AUC = 0.956, 95% CI: 0.919, 0.986; p‐tau217/Aβ42: AUC = 0.974, 95% CI: 0.948, 0.992) (Figure S2 and eTable 3).

In the classification of tau PET status, p‐tau217 outperformed Aβ42/40 and p‐tau181 in both the CADS (p‐tau217: AUC = 0.972, 95% CI: 0.951, 0.990; p‐tau181: AUC = 0.948, 95% CI: 0.916, 0.978, *p* < .05; Aβ42/40: AUC = 0.845, 95% CI: 0.786, 0.899, *p* < .05) and GHABS cohorts (p‐tau217: AUC = 0.935, 95% CI: 0.878, 0.976; p‐tau181: AUC = 0.909, 95% CI: 0.845, 0.963, *p* > .05; Aβ42/40: AUC = 0.802, 95% CI: 0.718, 0.875, *p* < .05) (Figure 2E–H and eTable 4). The ratio of p‐tau217/Aβ42 did not improve the AUC further (CADS: 0.974, 95% CI: 0.952, 0.992, *p* > .05; GHABS: 0.947, 95% CI: 0.900, 0.979, *p* > .05). Similar results were obtained when positive tau PET status was determined by temporal meta‐ROI CTR_z_ > 2 or mesial temporal CTR_z_ > 2 in the CADS cohort (Figure S2 and eTable 2) and temporal meta‐ROI SUVR ≥1.27 in the GHABS cohort in the sensitivity analysis (Figure S2 and eTable 3).

### 3.4 Head‐to‐head comparison of plasma and CSF biomarkers in classification of Aβ and tau PET status

**FIGURE 4:**
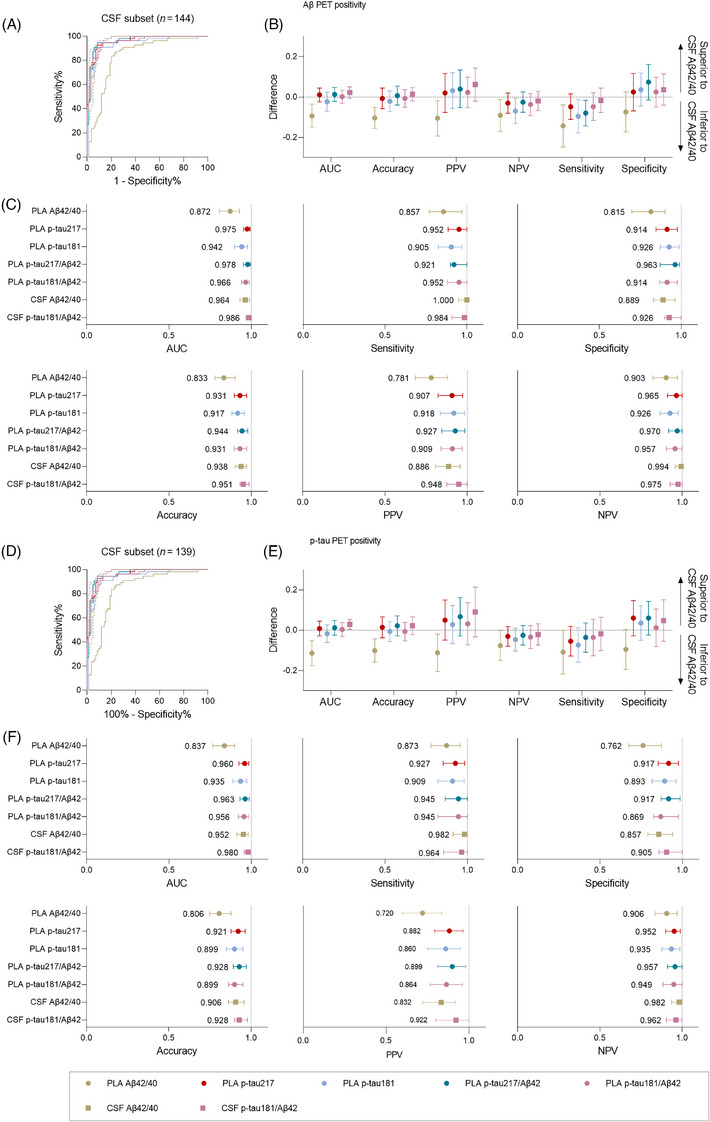
Head‐to‐head comparisons between plasma and CSF biomarkers for identifying Aβ and tau PET positivity in the CSF subset of CADS cohort. (A and D) ROC curves of plasma and CSF biomarkers for identifying Aβ (A) and tau (D) PET positivity by visual read. (B and E) Bootstrapped differences (*n* = 1000 resamples with replacement stratifying by output) in statistics between FDA‐approved CSF Aβ42/40 (reference) and other CSF and plasma biomarkers. The horizontal dashed line plotted at zero represents the lack of difference between CSF Aβ42/40 and other biomarkers. Other CSF and plasma biomarkers were considered to be clinically equivalent to CSF Aβ42/40 if the 95% CI of the mean difference included zero and clinically superior (>0) or inferior (<0) if it did not include zero. (C and F) AUC and other diagnostic metrics of plasma and CSF biomarkers for identifying Aβ (C) and tau (F) PET positivity. Dots and error bars represent the mean and 95% CI estimates from a bootstrapped sample. Aβ, amyloid beta; AUC, area under the curve; CI, confidence interval; CSF, cerebrospinal fluid; NPV, negative predictive value; PPV, positive predictive value; PLA, plasma; ROC, receiver operating characteristic.

To clarify the potential of blood biomarkers as standalone diagnostic tests, we conducted a head‐to‐head comparison of plasma and CSF biomarkers based on the Lumipulse platform in the CSF subset of the CADS cohort. In the classification of Aβ PET status (visual read), CSF Aβ42/40, which has been approved by the FDA for in vivo diagnosis of AD, showed a very high AUC of 0.964 (95% CI: 0.931, 0.989), whereas CSF p‐tau181/Aβ42 seemed to outperform CSF Aβ42/40, although the difference was not statistically significant (AUC = 0.986, 95% CI: 0.967, 0.999; *p* > .05). Interestingly, plasma p‐tau217 and the p‐tau217/Aβ42 ratio were clinically equivalent to the aforementioned CSF biomarkers (plasma p‐tau217: AUC = 0.975, 95% CI: 0.952, 0.993; p‐tau217/Aβ42: AUC = 0.978, 95% CI: 0.952, 0.996; *p* > .05) (Figure 4A–C and eTable 5).

In the classification of tau PET status, the performance of CSF p‐tau181/Aβ42 was superior to that of CSF Aβ42/40 (p‐tau181/Aβ42: AUC = 0.980, 95% CI: 0.957, 0.995; Aβ42/40: AUC = 0.952, 95% CI: 0.912, 0.981; *p* < .05). Additionally, plasma p‐tau217 and the p‐tau217/Aβ42 ratio were equivalent to those of both CSF Aβ42/40 and CSF p‐tau181/Aβ42 (p‐tau217: AUC = 0.960, 95% CI: 0.925, 0.985; p‐tau217/Aβ42: AUC = 0.963, 95% CI: 0.931, 0.988; *p* < .05) (Figure 4D–F and eTable 5). The bimodal distribution of CSF and blood biomarker levels was in good agreement with the classification of PET status (Figure S3).

### 3.5 Performance of blood and CSF biomarkers in the differential diagnosis of AD

Subsequently, we investigated the performance of plasma and CSF biomarkers for the diagnosis of AD versus non‐AD in patients with cognitive symptoms in the real‐world clinical setting. We first evaluated clinically relevant diagnostic metrics for plasma biomarkers in the entire CADS cohort, where AD is defined as clinically probable/possible AD or amnestic MCI with positive Aβ PET (visual read). When using the cutoffs for Aβ PET positivity based on the Youden index, plasma p‐tau217 and the p‐tau217/Aβ42 ratio showed overall accuracies of 91.6% and 92.3%, respectively, for distinguishing AD from non‐AD patients, with positive predictive values (PPVs) of 87.4% and 88.8% and negative predictive values (NPVs) of 95.5% for both (Figure S4 and eTable 6). Compared with CSF biomarkers in the CSF subset, plasma p‐tau217 alone performed equivalently to CSF Aβ40/42 and p‐tau181/Aβ42 ratios, with an overall accuracy of 91.3%, a PPV of 83.3%, and a NPV of 97.5% (CSF Aβ42/40: accuracy: 89.3%, PPV: 78.7%, NPV: 98.7%; CSF p‐tau181/Aβ42: accuracy: 92.0%, PPV: 83.6%, NPV: 98.8%; *p* > .05), whereas the plasma p‐tau217/Aβ42 ratio showed a comparable diagnostic performance to that of p‐tau217 alone (p‐tau217/Aβ42: accuracy: 92.7%, PPV: 88.3%, NPV: 95.5%) (eTable 7).

Given that several neurodegenerative diseases may be comorbid with Aβ or tau pathology, AD was also defined as A+T+ regardless of the clinical diagnosis in terms of its biological definition. Thus, we also evaluated the performance of plasma and CSF biomarkers for identifying individuals with A+T+ versus others (A−T−, A−T+, A+T−) in CADS. Similarly, p‐tau217 alone and the p‐tau217/Aβ42 ratio performed best among all the plasma biomarkers (p‐tau217: accuracy: 95.3%, PPV: 91.9%, NPV: 98.1%; p‐tau217/Aβ42: accuracy: 94.8%, PPV: 91.7%, NPV: 97.2%) (Figure S4 and eTable 6). Compared with CSF biomarkers in the CSF subset, plasma p‐tau217 and p‐tau217/Aβ42 performed equivalently to CSF p‐tau181/Aβ42 and Aβ42/40 ratios in identifying biological AD (A+T+) (plasma p‐tau217: accuracy: 91.0%; PPV: 83.6%; NPV: 97.3%; plasma p‐tau217/Aβ42: accuracy: 93.2%; PPV: 89.2%; NPV: 96.2%; CSF Aβ42/40: accuracy: 90.2%; PPV: 80.4%; NPV: 100%; CSF p‐tau181/Aβ42: accuracy: 93.2%; PPV: 85.5%; NPV: 100%; *p* > .05) (eTable 7).

### 3.6 Two‐cutoff approach improves diagnostic performance of plasma biomarkers

To further improve the diagnostic performance of plasma biomarkers as standalone diagnostic tests, we applied the two‐cutoff approach to stratify individuals into three categories: clearly normal, clearly abnormal, and uncertain individuals (intermediate) requiring further confirmatory tests.

**FIGURE 5:**
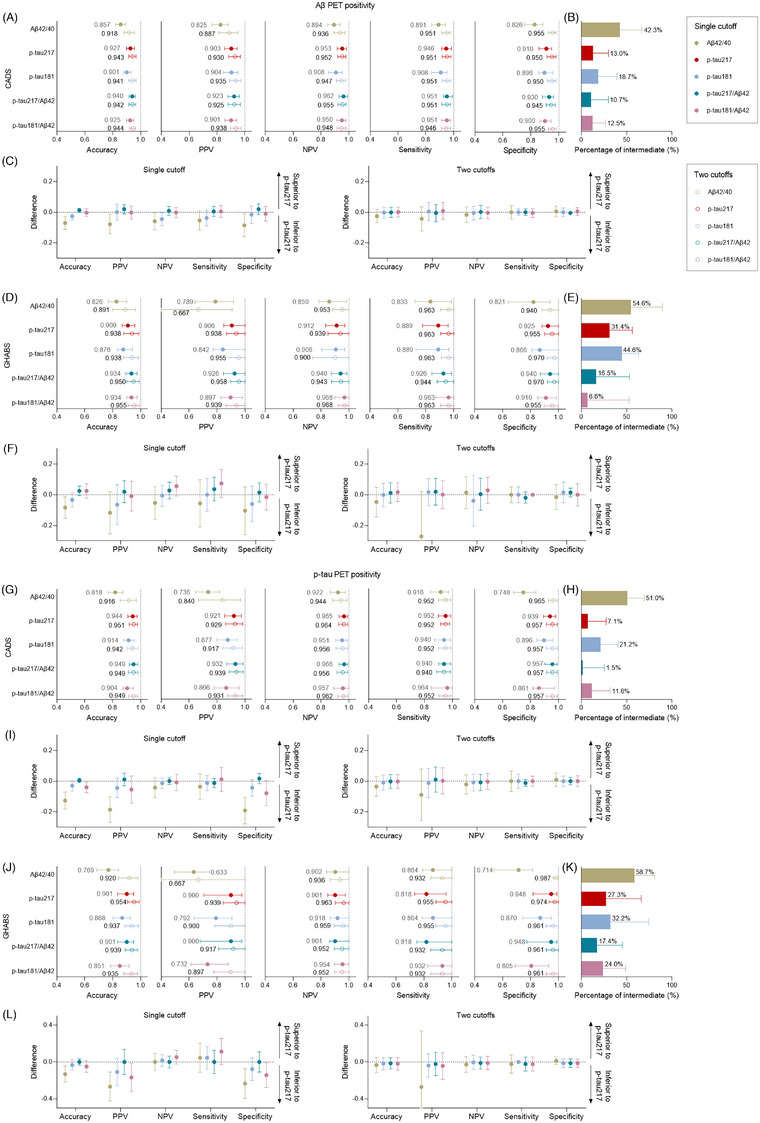
The two‐cutoff approach improved the diagnostic performance of plasma biomarkers in the entire CADS and GHABS cohorts. (A, D, G, J) Diagnostic metrics of plasma biomarkers for identifying Aβ (A and D) and tau (G and J) PET positivity in CADS (A and G) and GHABS (D and J) cohorts by the single‐cutoff and two‐cutoff approaches, respectively. In the single‐cutoff approach, the threshold was calculated using the maximum Youden index. In the two‐cutoff approach, the lower threshold was obtained by maximizing specificity with sensitivity fixed at 95%, whereas the upper threshold was obtained by maximizing sensitivity while fixing specificity at 95%. Dots and error bars represent the mean and 95% CI estimates from a bootstrapped sample. (B, E, H, K) Percentages (with a 95% CI estimate from a bootstrapped sample) of participants who fell into the gray zone between two cutoffs were classified in the intermediate group. (C, F, I, L) Bootstrapped differences (*n* = 1000 resamples with replacement stratifying by the output) in the statistics between plasma p‐tau217 (reference) and other plasma biomarkers for both the single‐cutoff and two‐cutoff approaches. The horizontal dashed line plotted at zero represents the lack of difference between plasma p‐tau217 and other biomarkers. Other plasma biomarkers were considered to be clinically equivalent to p‐tau217 if the 95% CI of the mean difference included zero and clinically superior (>0) or inferior (<0) if it did not include zero. CADS, Chongqing Ageing & Dementia Study; CI, confidence interval; CSF, cerebrospinal fluid; GHABS, Greater‐Bay‐Area Healthy Aging Brain Study NPV, negative predictive value; PPV, positive predictive value; p‐tau, phosphorylated tau.

As mentioned earlier, plasma p‐tau217 and p‐tau217/Aβ42 exhibited excellent performance. In the classification of Aβ PET status in the entire CADS cohort, applying the two‐cutoff approach further increased the specificity without decreasing its sensitivity for both p‐tau217 and p‐tau217/Aβ42 (p‐tau217: specificity: 95 .0% vs 91.0%, sensitivity: 95 .1% vs 94.6%; p‐tau217/Aβ42: specificity: 94 .5% vs 93.0%, sensitivity: 95 .1% vs 95.1%). Importantly, p‐tau217/Aβ42 had a lower percentage of intermediate cases than p‐tau217 alone (10.7% vs 13.0%) (Figure 5A–C and eTable 1). In the classification of tau PET status, applying the two‐cutoff approach improved the overall accuracy and specificity to 95.1% and 95.7% for p‐tau217 with an intermediate percentage of 7.1%, and to 94.9% and 95.7% for p‐tau217/Aβ42 with an intermediate percentage of 1.5% (Figure 5G–I and eTable 4). For identifying clinically diagnosed AD and biological AD, using a two‐cutoff approach improved the diagnostic accuracy to 94.1% to 96.4% for plasma p‐tau217 and to 93.4% to 96.0% for p‐tau217/Aβ42, with intermediate percentages of 10.9% to 12.8% for p‐tau217 and 8.9% to 10.5% for p‐tau217/Aβ42 (Figure S4, eTables 6 and 7).

In GHABS, under the two‐cutoff approach, plasma p‐tau217 had an improved overall accuracy of 93.8%, with 96.3% sensitivity, 95.5% specificity, and 31.4% intermediate percentages for identifying Aβ positivity, as well as 95.4% accuracy, 95.5% sensitivity, 97.4% specificity, and 27.3% intermediate percentages for identifying tau PET positivity (Figure 5D–F and J–L, eTables 1 and 4). Plasma p‐tau217/Aβ42 showed the same high accuracy with p‐tau217 using the two‐cutoff approach but had a reduced intermediate percentage (16.5% vs 31.4% for Aβ positivity, 17 .4% vs 27.3% for tau positivity). Similar results were obtained using quantitatively determined Aβ and tau PET statuses in the sensitivity analysis (eTables 2 and 3).

We then analyzed the concordance of plasma and CSF biomarkers in classifying Aβ PET status using the two‐cutoff approach. The overall agreement of plasma p‐tau217 and p‐tau217/Aβ42 with CSF Aβ42/40 in the classification of Aβ PET status was 79% to 82%, 85% to 86%, and 70% to 77%, respectively, in the total, negative, and positive subgroups and the agreement of plasma tests with CSF p‐tau181/Aβ42 was 89% to 91%, 88% to 90%, and 91% to 93% (Figure S5).

## 4 DISCUSSION

In routine diagnostic workups in real‐world clinical settings, it is often difficult for clinicians to separate AD and other causes of cognitive decline because of overlapping symptoms, but the principles of treatment for these conditions are different. Even when patients are evaluated by dementia specialists, the aetiologic diagnoses change in 20% to 36% of patients, and management strategies change in 36.5% to 63.5% of patients following amyloid PET scans.^3^, ^4^ These figures would be much greater in the primary care setting. Blood tests have become the research hotspot due to their advantages of cost‐effectiveness and convenience.

Lumipulse plasma p‐tau217 used in this study is a novel blood test with better clinical utility, with the reagents individually packaged for single use and higher reliability. Lumipulse plasma p‐tau217/Aβ42 ratio has been filed with the FDA for the in vitro diagnosis of AD. In this study, using Aβ and tau PET as the classification standards, we demonstrated the high performance of both plasma p‐tau217 alone (AUC values of 0.940 to 0.960 and 0.935 to 0.972, respectively) and p‐tau217/Aβ42 ratio (AUC values of 0.963 to 0.966 and 0.947 to 0.974, respectively) in both clinical and community cohorts. The performance of Lumipulse plasma Aβ42/40 (AUC = 0.867 to 0.888) also on discriminating positive from negative Aβ PET status was similar to previously reported performance^25^, ^26^, ^36^; however, plasma p‐tau181 performed better in this study than in previous studies, with slightly lower AUC values of 0.916 to 0.942 and 0.909 to 0.948 respective to p‐tau217 for identifying Aβ and tau PET positivity, respectively.^37^ In the head‐to‐head comparisons, plasma p‐tau217 and p‐tau217/Aβ42 performed equivalently to the CSF Aβ42/40 and p‐tau181/Aβ42 ratios in determining Aβ and tau PET positivity and diagnosing AD. These findings indicate that plasma p‐tau217 and p‐tau217/Aβ42 may be able to replace CSF and PET measures in the diagnostic workup of AD. Importantly, our study found that plasma p‐tau217/Aβ42 ratio performed better than p‐tau217 alone, and the difference was meaningful in the community cohort but not in the clinical cohort.

**FIGURE 6 alz70038-fig-0006:**
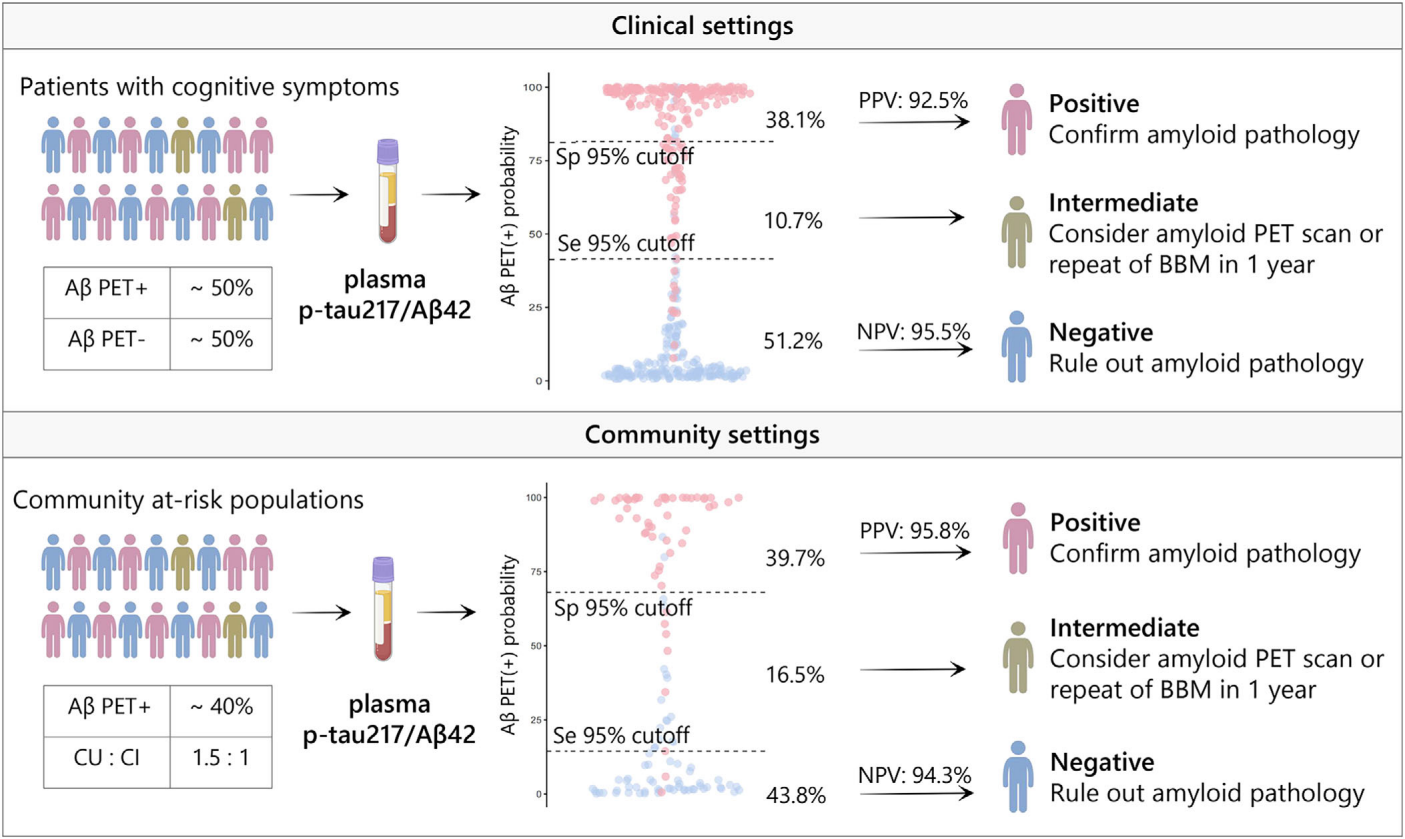
The two‐step workflow of predicting amyloid pathology based on plasma p‐tau217/Aβ42 ratio in clinical and community settings. Use of two cutoff values for Lumipulse plasma p‐tau217/Aβ42 test in patients with cognitive symptoms in clinics and in community‐based at‐risk populations leads to three categories of results: positive, intermediate, and negative, increasing the accuracy with which people can be classified as having or not having amyloid pathology. BBM, blood‐based biomarker; CI, cognitively impaired; CU, cognitively unimpaired; NPV, negative predictive value; PPV, positive predictive value; p‐tau, phosphorylated tau; Se, sensitivity; Sp, specificity.

The proposed two‐step workflow increases the diagnostic performance of plasma biomarkers and expands their practicability and generalizability.^38^ As expected, the two‐cutoff approach increased specificity; still, it did not decrease the sensitivity of plasma p‐tau217 and p‐tau217/Aβ42 ratio, thereby increasing the overall accuracy in classifying Aβ and tau PET statuses, as well as in diagnosing AD (Figure 6 and Figure S6). The intermediate percentages of plasma p‐tau217 in identifying Aβ PET status in our clinical (13.0%) and community cohort (31.4%) were similar to those previously reported when 95% sensitivity and 95% specificity were used to determine two cutoffs.^22^, ^23^, ^38^ Similarly, the plasma p‐tau217/Aβ42 ratio reduced the intermediate percentages from 13.0% to 10.7% in the clinical cohort and from 31.4% to 16.5% in the community cohort, and the reduction was meaningful in the latter. Taken together, our findings indicate that the superiority of the p‐tau217/Aβ42 ratio is more pronounced in populations at an early stage of AD or when the prevalence of amyloid is expected to be low as in CU subjects (60% CU in GHABS vs 11% CU in CADS).

According to the minimum acceptable performance of blood tests of amyloid pathology for clinical use recommended by the Global CEO Initiative on AD,^39^ for use as a confirmatory test without follow‐up tests, a blood test should have performance equivalent to that of CSF tests with a sensitivity and specificity of ≥90% and a PPV and NPV of ≥90% when the Aβ prevalence is ∼50%, which is close to the true percentage (48%) in patients with cognitive symptoms in our clinical setting. In addition, the percentage of intermediate individuals when using a two‐cutoff approach should be < 15% to 20%. Plasma p‐tau217 alone and the p‐tau217/Aβ42 ratio fully met the aforementioned acceptable criteria in this study. Our and other groups’ studies demonstrated the clinical utility of blood tests, which may be used as standalone in the diagnostic workup of AD and largely reduce the reliance on PET scans or CSF tests, thereby reducing the operation and economic burden.

In our study, we used visual read results as the primary outcomes of Aβ and tau PET statuses because visual assessment is the most commonly used method in clinical practice and has been approved by the FDA and the European Medicines Agency (EMA). In the sensitivity analyses, we also included the quantitative results of PET as an additional outcome. In CADS, the accuracies of plasma p‐tau217 and p‐tau217/Aβ42 were also high, with AUC values ranging from 0.927 to 0.951 when quantitative PET assessment was used as the outcome, but these values were slightly inferior to those when visual results were used. This may be because the PET image analysis was spatially normalized via the MR‐less CapAIBL approach, which could reduce the heterogeneity caused by different MRI scanners with very different resolutions and parameters and has wider generalizability but at the expense of some precision in terms of spatial location.^40^ In GHABS, the AUC of plasma p‐tau217 and p‐tau217/Aβ42 was greater, ranging from 0.956 to 0.974 using quantitative PET outcome than visual read outcome (0.935 to 0.963). This may be because the majority of participants in the GHABS cohort were in the early stage of AD with intact cognition, when some Aβ or tau PET images were indistinguishable for visual assessment, especially for the tau tracer AV1451.[Fig alz70038-fig-0006]

In the clinic cohort of this study, all patients with cognitive symptoms or cognitive concerns who were willing to participate in this study were consecutively recruited. We did not exclude potential participants due to other diseases to maintain a real‐world clinical setting as much as possible. Thus, the cutoffs of blood biomarkers in this study are suitable for clinical practice. However, validation in a prospective cohort with this preset cutoff is needed. Notably, interpreting blood test results in patients should not be isolated from the clinical context. On the one hand, some other neurodegenerative diseases or conditions often involve comorbid Aβ or tau pathology. For example, it has been reported that the prevalence of amyloid deposition measured by CSF Aβ42/40 or PET has been reported to be 47% in patients with DLB^41^; this prevalence was 37.5% in our study. On the other hand, soluble Aβ and tau in the blood can be metabolized by peripheral tissue and organs; thus, blood tests could be affected by comorbidities, such as chronic kidney disease, hypertension, a history of myocardial infarction, or stroke.^42^, ^43^, ^44^, ^45^ Whether the p‐tau217/Aβ42 ratio has an advantage in reducing the interference of comorbidities remains to be explored in future studies. However, in the community cohort in this study, individuals with no signs or concerns about AD were not willing to undergo PET scans during the recruitment period. Thus, GHABS participants included in this study may be at higher risk or probability for AD and could not reflect the population characteristics in the real‐world community. Additionally, the average participants, aged 66.2 in CADS and 66.6 in GHABS, are relatively younger than the typical age of late 70s, suggesting that some early‐onset AD patients with higher levels of amyloid and tau pathology were also included in this study. In addition, many individuals over 80 years old refused to participate in the study because they were not willing to undergo PET scan or lumbar puncture. Also, more than 50% of participants in our clinical cohort were non‐AD dementia, whose age at onset is relatively earlier. These reasons together lead to our younger cohort. Last but not least, in this study, the levels of plasma biomarkers differed between the two cohorts, hampering the establishment of a common cutoff. Therefore, before translation into clinical practice, there is an urgent need for a unified standard operating procedure to control the preanalytical and analytical factors, as well as certified reference materials and methods to standardize the assays at different times or different centers.^46^, ^47^ As a member of the Alzheimer's Association Quality Control Program for Blood and CSF biomarkers, we adopt the international standard operating procedures for CSF and blood sample collection and processing,^48^ laying the foundation for the high performance of CSF and blood tests in the present study.

In conclusion, this study highlights the clinical utility of the plasma p‐tau217/Aβ42 ratio in determining cerebral amyloid and tau accumulation statuses with an overall accuracy of approximately 95% when implementing the two‐cutoff approach, which is clinically equivalent to FDA‐approved CSF tests, in patients with cognitive symptoms in clinics and in community‐based cohorts with or without cognitive impairment. The clinical application of these blood tests would substantially reduce the reliance on PET or CSF tests by approximately 90% in clinical settings and approximately 84% in community settings, thereby enhancing access to accurate AD diagnosis in clinics and reducing the economic burden. This study will pave the way for translating blood‐based biomarkers from research studies to clinical practice.

## AUTHOR CONTRIBUTIONS

J.W., S.H., Y.J.L., and Y.L.G contributed equally as first authors. Y.J.W., T.F.G., H.Z., and C.L.M. jointly supervised this work. Y.J.W., J.W., Y.T., J.T.Y., and Q.C. conceived the project. J.W. and S.H. performed CSF and blood tests. Q.H.W. and S.H. analyzed the data. P.B., V.D., and J.F. performed a quantitative analysis of PET imaging from CADS. X.C. performed the visual reads of PET images. Y.J.W. provided mentorship and founded and led the CADS study, which enabled the recruitment of participants in this study. Y.J.L., X.L.B., Y.H.L., Y.C., and F.Z. made the diagnosis. T.F.G. provided mentorship and founded and led the GHABS study, which enabled the recruitment of participants in this study. G.Y.L., L.H.Z., and A.Q. L. analyzed data from the GHABS cohort. Y.C., P.S., and Z.B.Z. performed the quantitative and visual analysis of PET imaging from the GHABS study. J.W. and Y.J.W. wrote the initial draft of the manuscript. All authors made substantial contributions to subsequent versions of the manuscript and approved the final version for submission.

## CONFLICT OF INTEREST STATEMENT

H.Z. has served on scientific advisory boards and/or as a consultant for AbbVie, Acumen, Alector, Alzinova, ALZPath, Amylyx, Annexon, Apellis, Artery Therapeutics, AZTherapies, Cognito Therapeutics, CogRx, Denali, Eisai, LabCorp, Merry Life, Nervgen, Novo Nordisk, Optoceutics, Passage Bio, Pinteon Therapeutics, Prothena, Red Abbey Labs, reMYND, Roche, Samumed, Siemens Healthineers, Triplet Therapeutics, and Wave and has given lectures at symposia sponsored by Alzecure, Biogen, Cellectricon, Fujirebio, Lilly, Novo Nordisk, and Roche. The other authors declare no competing interests. Author disclosures are available in the Supporting information.

## Supporting information



Supporting Information

Supporting Information

Supporting Information
