# Supplementary material for: Diagnostic accuracy of plasma p‐tau217/Aβ42 for Alzheimer's disease in clinical and community cohorts
Source: Alzheimers Dement. 2025 Mar 29;21(3):e70038. doi: 10.1002/alz.70038 (PMC11953589; doi:10.1002/alz.70038)
Supplement: Supplementary file 3 — Supporting Information [file ALZ-21-e70038-s003.docx]

**Title:**

Diagnostic accuracy of plasma p-tau217/Aβ42 for Alzheimer’s disease in clinical and community cohorts

**Authors:**

Jun Wang, PhD^1,2,#^, Shan Huang, PhD^1,2,#^, Guoyu Lan, PhD^3,#^, Yu-Jie Lai, MD^1,2,#^, Qing-Hua Wang, PhD^1,2^, Yang Chen, PhD^1,2^, Zhong-Song Xiao, MD^4^, Xiao Chen, PhD^5^, Xian-Le Bu, PhD^1,2^, Yu-Hui Liu, PhD^1,2^, Fan Zeng, PhD^1,2^, Laihong Zhang, Ms^3^, Anqi Li, Ms^3^, Yue Cai, PhD^3^, Pan Sun, PhD^3^, Zhengbo He, Mr^3^, Vincent Doré, PhD^6.7^, Jurgen Fripp, PhD^6^, Pierrick Bourgeat, PhD^6^, Qin Chen, PhD^8^, Jin-Tai Yu, PhD^9^, Yi Tang, PhD^10^, Henrik Zetterberg, PhD^11,12,13,14,15,16^, Colin L. Masters, MD^17^, Tengfei Guo, PhD^3,*^, Yan-Jiang Wang, PhD^1,2,18,*^, for the Translational Biomarker Research of AgIng and Neurodegeneration (TBRAIN)

**Supplemental Materials:**

**Figure S1.** Correlations of plasma and CSF biomarkers in CSF subset of CADS cohort.

**Figure S2.** Performance of plasma biomarkers in classification of Aβ and tau PET status by quantitative results.

**Figure S3.** Frequency distribution of plasma and CSF biomarkers in the CSF subset of CADS cohort.

**Figure S4.** Performance of plasma biomarkers in diagnosis of AD in CADS cohort.

**Figure S5.** Concordance of plasma and CSF biomarkers in the classification of Aβ PET status in the CSF subset of CADS.

**Figure S6.** The two-step workflow of predicting amyloid pathology based on plasma p-tau217 test in the clinical and community settings.

**Table S1.** Concordance rate of Aβ PET status between visual read and quantitative results.

**Table S2.** Variability of CSF and plasma biomarkers measured on Lumipulse G1200 platform in quality control.

**Table S3.** Diagnosis of cognitively impaired participants in CADS cohort.

**Table S4.** Correlations of plasma biomarkers with Aβ and tau PET in individuals with two PETs in CADS.

**eTable 1.** Accuracy of plasma biomarkers in classification of Aβ PET status by visual read in the entire CADS and GHABS cohorts.

**eTable 2.** Accuracy of plasma biomarkers in classification of Aβ and tau PET statuses by quantitative results in the entire CADS cohort in sensitivity analysis.

**eTable 3.** Accuracy of plasma biomarkers in classification of Aβ and tau PET statuses by quantitative results in GHABS cohort in sensitivity analysis.

**eTable 4.** Accuracy of plasma biomarkers in classification of Tau PET status by visual read in the entire CADS and GHABS cohorts.

**eTable 5.** Head-to-head comparison of plasma and CSF biomarkers in classification of Aβ and tau PET statuses by visual read in the CSF subset of CADS cohort.

**eTable 6.** Accuracy of plasma biomarkers in diagnosis of AD.

**eTable 7.** Head-to-head comparison of plasma and CSF biomarkers in diagnosis of AD in the CSF subset of CADS cohort.

**
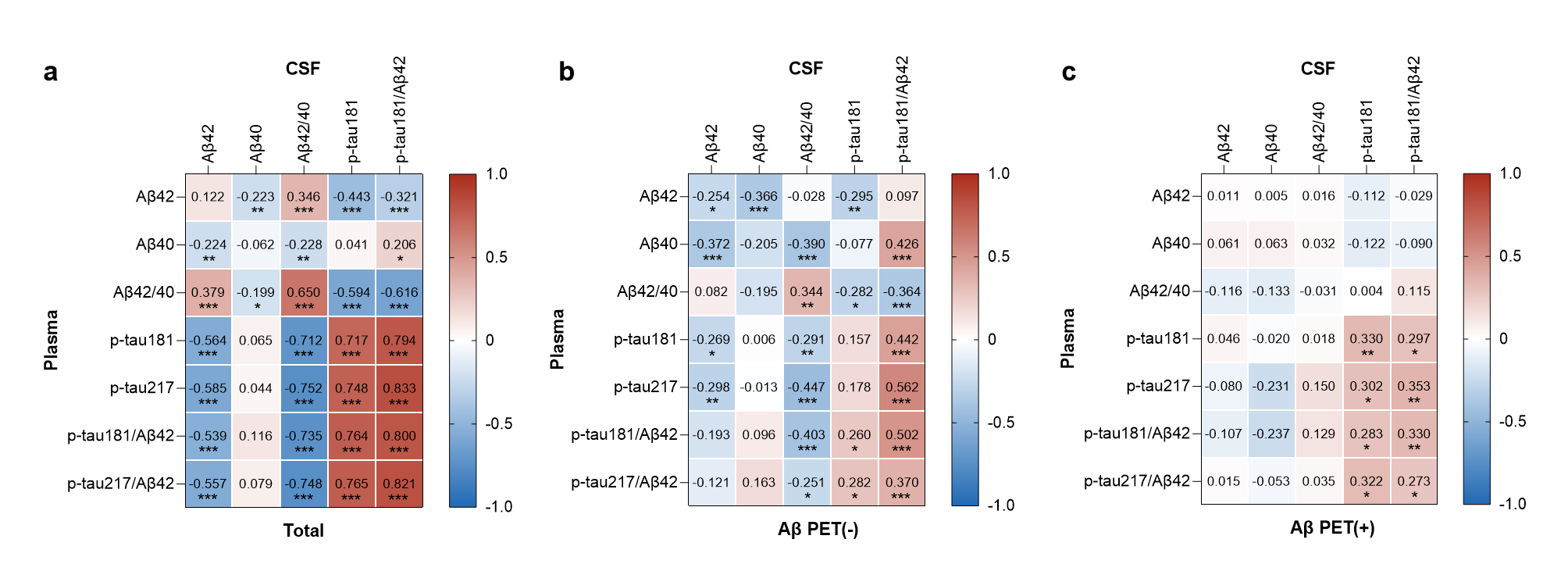
**

**Figure S1. Correlations of plasma and CSF biomarkers in CSF subset of CADS cohort.**

The heatmap shows the spearman ρ of correlations between plasma and CSF biomarkers. CSF, cerebrospinal fluid. *, p<0.05; **, p<0.01; ***, p<0.001.


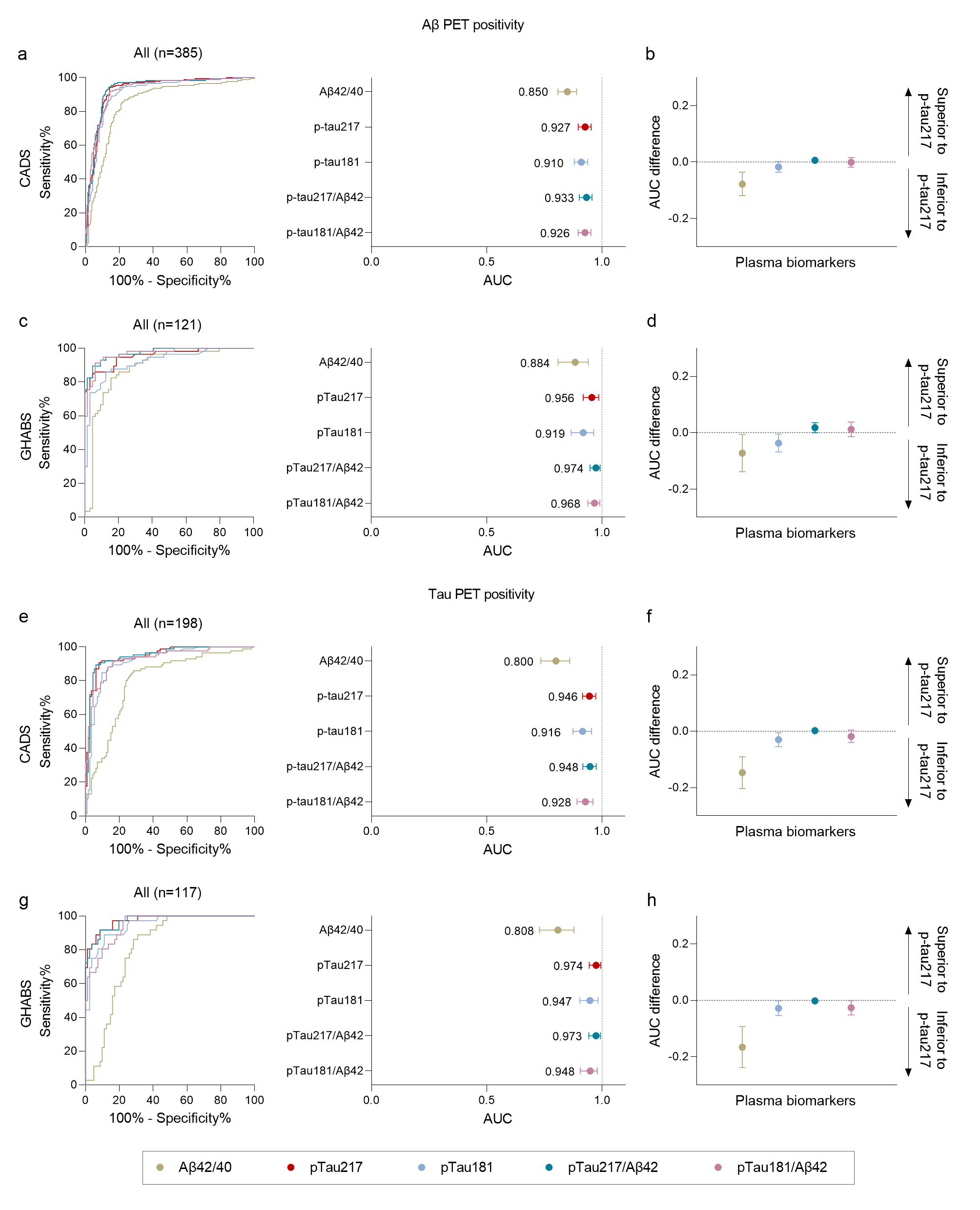


**Figure S2. Performance of plasma biomarkers in classification of Aβ and tau PET status by quantitative results. a,c,e,g,** ROC curve and AUC of plasma biomarkers in classification of Aβ **(a,c)** and tau PET **(e,g)** status in the entire CADS **(a,e)** and GHABS **(c,g)** cohorts. In CADS, Aβ PET positivity was defined as centiloids>25, while tau PET positivity as mesial temporal CTR_z_ >2. In GHABS, Aβ PET positivity were defined as D3FSP COMPOSITE SUVR ≥0.78 and AV45 SUVR ≥ 1.11; while tau PET positivity were defined as temporal meta-ROI MK-6240 SUVR≥1.27. Vertical dashed lines represent the AUC = 1. **b,d,f,h,** Bootstrapped differences (n = 1,000 resamples with replacement stratifying by the output) between the statistics using plasma p-tau217 (reference) and other plasma biomarkers in CADS **(b,f)** and GHABS **(d,h)** cohorts. The horizontal dashed line plotted at zero represents the lack of difference between plasma p-tau217 and other plasma biomarkers. Other plasma biomarkers were considered to be clinically equivalent to p-tau217 if the 95% CI of the mean difference included zero and clinically superior (>0) or inferior (<0) if it did not include zero. Dots and error bars represent the actual statistic and 95% CI (from bootstrapped n = 1,000 samples with replacement), respectively. AUC, area under the curve; CI, confidence interval.

**
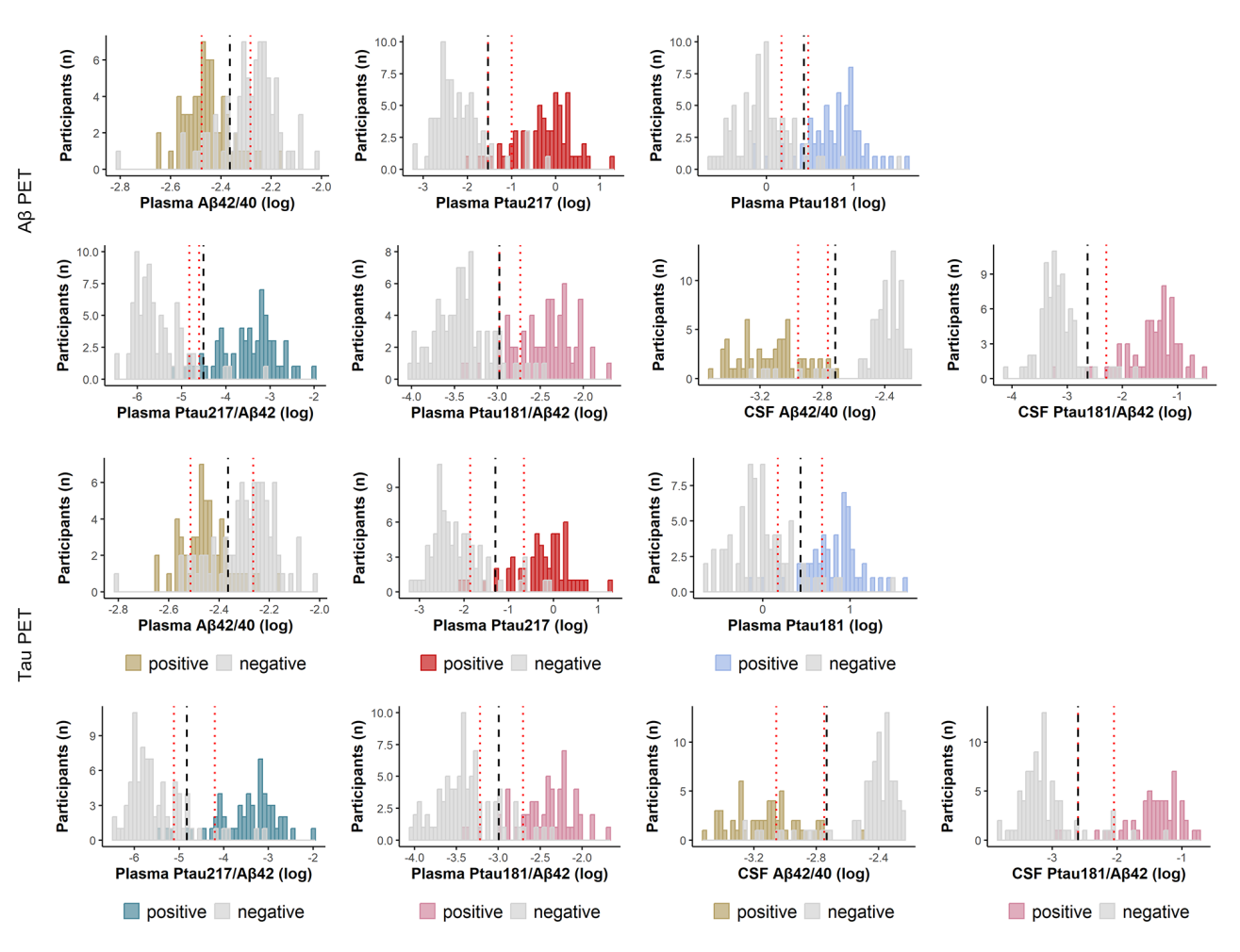
**

**Figure S3.** **Frequency distribution of plasma and CSF biomarkers in the CSF subset of CADS cohort.** Histograms represent the distribution of the data colored by Aβ and tau PET imaging status. The vertical black line represents the threshold derived from the single-cutoff approach, and red lines represent the lower and upper thresholds from the two-cutoffs approach. Aβ and tau PET positivity were assessed by visual read method.


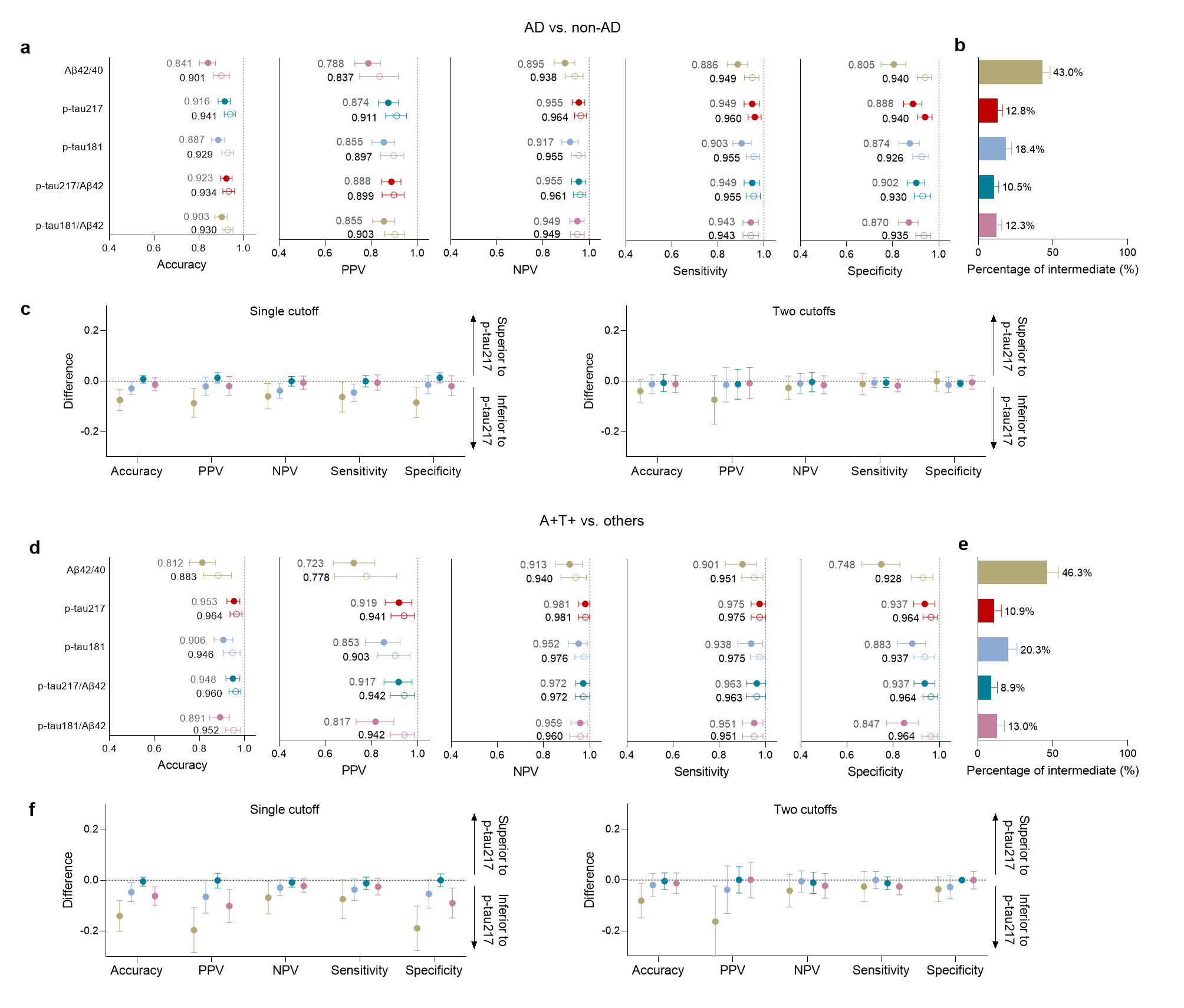


**Figure S4. Performance of plasma biomarkers in diagnosis of AD in CADS cohort.** Diagnostic metrics of plasma biomarkers in diagnosis of clinical **(a-c)** and biological AD **(d-f)** by single-cutoff and two-cutoffs approach respectively. Clinical AD is defined as probable/possible AD or amnesic MCI with positive Aβ PET (visual read), while biological AD is defined as A+T+ regardless of clinical diagnosis.


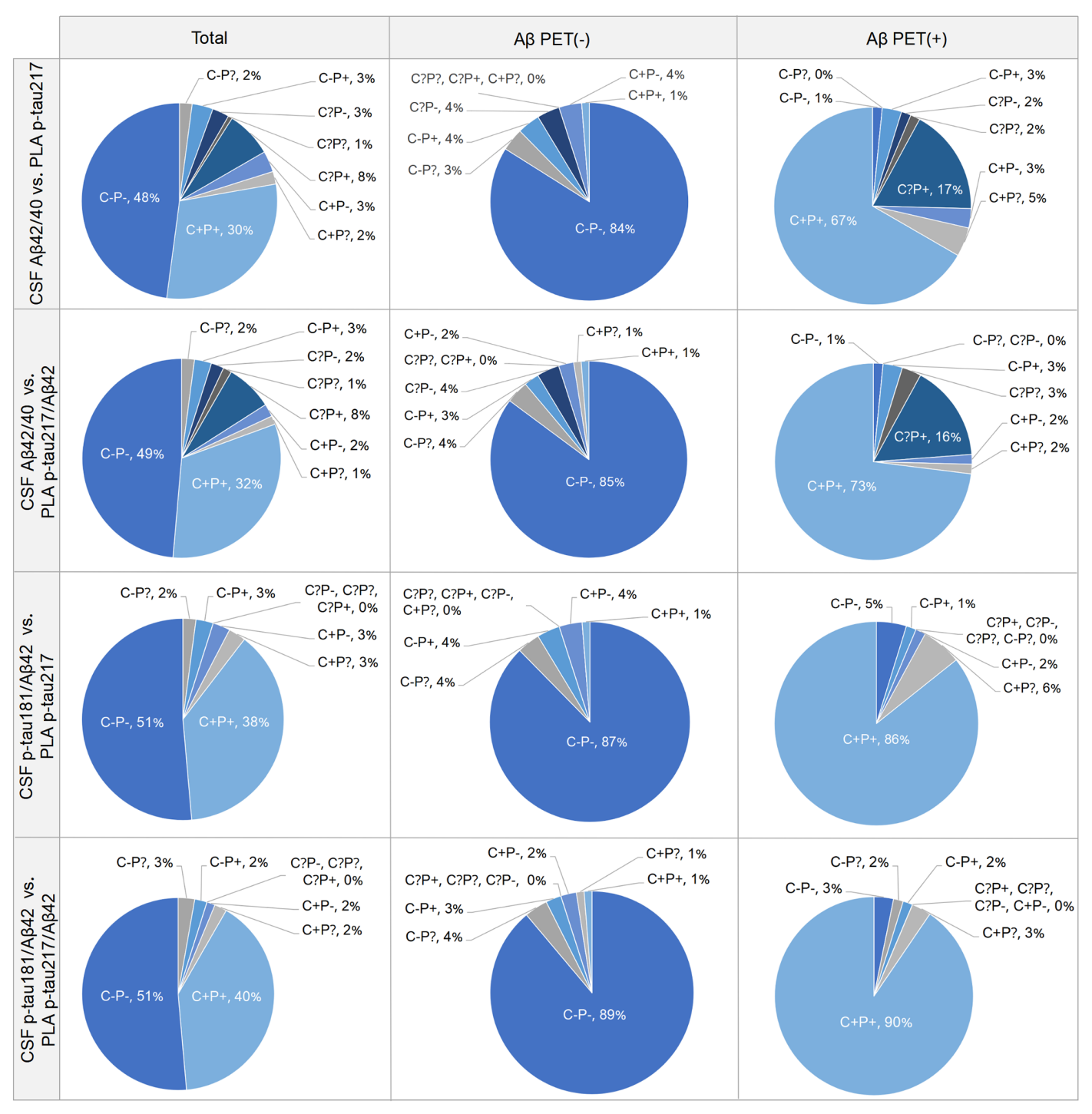


**Figure S5. Concordance of plasma and CSF biomarkers in the classification of Aβ PET status in the CSF subset of CADS.** CSF (C) and plasma (P) biomarkers were respectively classified into three categories based on the two-cutoffs approach: positive (+), negative (-), and intermediate (?).

**
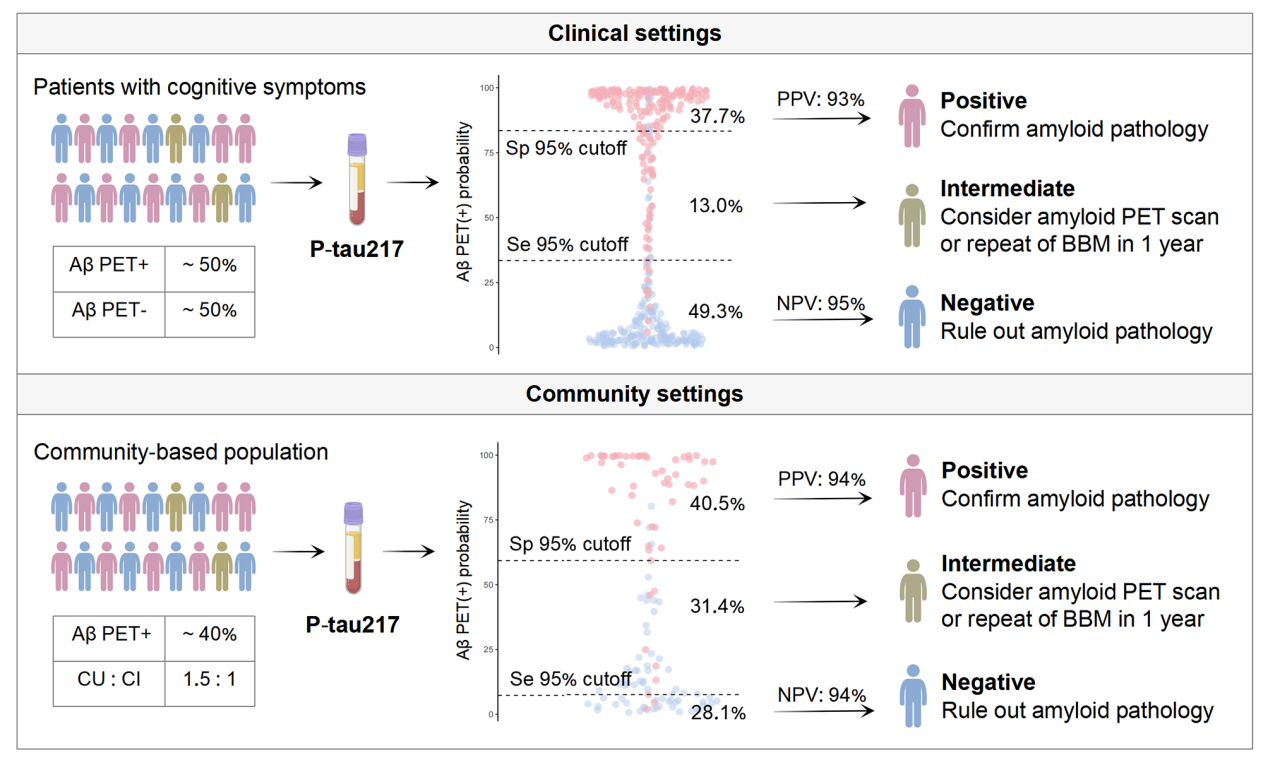
Figure S6. The two-step workflow of predicting amyloid pathology based on plasma p-tau217 test in the clinical and community settings.** Use of two cut-off values for Lumipulse plasma p-tau217 test in patients with cognitive symptoms in clinics and in community-based population leads to three categories of results: positive, intermediate and negative, increasing the accuracy with which people can be classified as having or not having amyloid pathology. BBM, blood-based biomarkers; CI, cognitively impaired; CU, cognitively unimpaired; NPV, negative predictive value; PPV, positive predictive value; Se, sensitivity; Sp, specificity.

**Table S1. Concordance rate of Aβ PET status between visual read and quantitative results.**

| CADS cohort | | | | |
| --- | --- | --- | --- | --- |
|  | Visual read: negative (n=201) | | Visual read: positive (n=184) | |
|  | No. | Percentage (%) | No. | Percentage (%) |
| Single cutoff | | | | |
| CL<25 | 195 | 97.0% | 16 | 8.7% |
| CL≥25 | 6 | 3.0% | 168 | 91.3% |
| Two cutoffs | | | | |
| CL<15 | 188 | 93.5% | 8 | 4.3% |
| CL: 15-35 | 10 | 5.0% | 22 | 12.0% |
| CL>35 | 3 | 1.5% | 154 | 83.7% |
| GHABS cohort | | | | |
|  | Visual read: negative (n=67) | | Visual read: positive (n=54) | |
|  | No. | Percentage (%) | No. | Percentage (%) |
| SUVR: negative | 62 | 92.5% | 2 | 3.7% |
| SUVR: positive | 5 | 7.5% | 52 | 96.3% |

The total concordance rate between visual read and quantitative results was 94.3% in CADS and 94.2 in GHABS using the single cutoff approach. In GHABS, Aβ PET positivity were defined as D3FSP COMPOSITE SUVR ≥0.78 and AV45 SUVR ≥1.11. Abbreviation: CL, Centiloid; SUVR, standard uptake value ratio.

**Table S2. Variability of CSF and plasma biomarkers measured on Lumipulse G1200 platform in quality control.**

| Variabilities | | CSF biomarker assay | | | Plasma biomarker assay | | | |
| --- | --- | --- | --- | --- | --- | --- | --- | --- |
|  |  | Aβ42 | Aβ40 | p-tau181 | Aβ42 | Aβ40 | p-tau181 | p-tau217 |
| LLoQ (pg/mL) | | 9 | 5 | 1.1 | 0.43 | 0.44 | 0.261 | 0.037 |
| Quantifiable samples (%) | | 100 | 100 | 100 | 100 | 100 | 100 | 100 |
| Duplicate CVs (%) | Lumipulse controls | 0.633 (0-2.00) | 0.827 (0.014-3.81) | 1.24 (0-5.72) | 0.558 (0.004-4.15) | 0.636 (0.073-3.45) | 1.37 (0.124-3.79) | 2.00 (0-3.86) |
|  | In-house controls | 2.49 (0.141-8.07) | 2.04 (0.129-8.41) | 1.71 (0.252-5.90) | 1.40 (0.221-4.51) | 0.761 (0.039-4.51) | 1.90 (0-4.29) | 3.12 (0.559-9.95) |
| Longitudinal CVs (%) | Lumipulse controls | 1.23 (1.02-1.34) | 1.02 (0.97-1.86) | 2.79 (2.75-4.23) | 6.52 (4.59-8.45) | 3.68 (2.70-4.66) | 3.52 (3.36-3.67) | 2.52 (1.59-3.45) |
|  | In-house controls | 4.68 (4.30-5.17) | 5.07 (4.08-5.26) | 2.99 (2.19-3.13) | 5.05 （3.65-4.97） | 1.62 (0.650-2.26) | 5.31 (2.66-6.58) | 5.85 (5.09-7.74) |

Data are stated as median (range). There are 3 Lumipulse controls and 3 in-house control samples for CSF assay, as well as 2 Lumipulse controls and 4 in-house control samples for plasma assay. Quantifiable samples means that samples with biomarker concentrations above the LLoQs. Duplicate CVs: There were 33 replicate measurements of Lumipulse controls and in-house control samples for each CSF biomarker separately, and one outlier with an abnormally high value was removed for CSF Aβ42 and Aβ40 in-house control samples. There were 16 replicate measurements of Lumipulse controls and 14 replicate measurements of in-house control samples for plasma biomarkers, and one outlier with an abnormally high value was removed for plasma p-tau181 and p-tau217 in-house control samples. Measurement period of longitudinal CVs : CSF biomarkers: 2024.2-2024.5 (11 rounds); Plasma biomarkers: 2024.3-2024.4 (14 rounds for Lumipulse controls; 11 rounds for in-house control samples). CV, Coefficients of variation; LLoQ, the lower limit of quantification; LLoQ (CV): plasma p-tau181 (10%), other biomarkers (20%).

**Table S3. Diagnosis of cognitively impaired participants in CADS cohort.**

| **Diagnosis** | **No. (%)** |
| --- | --- |
| Alzheimer’s disease with dementia | 130 (33.25%) |
| Mild cognitive impairment due to Alzheimer’s disease | 46 (11.76%) |
| Vascular cognitive impairment | 29 (7.42%) |
| Parkinson’s disease with or without cognitive impairment | 14 (3.58%) |
| Atypical parkinsonion disorders (dementia with Lewy bodies, progressive supranuclear palsy, multiple system atrophy, corticobasal syndrome) | 34 (8.70%) |
| Other neurodegenerative diseases (e.g. frontotemporal dementia, primary progressive aphasia, motor neuron disease, neuronal intranuclear inclusion disease, etc) | 30 (7.67%) |
| Other non-neurodegenerative disorders (e.g. hydrocephalus, metabolic disorders, autoimmune encephalitis, neurosyphilis, mood or sleep disorders, etc） | 51 (13.04%) |
| Cognitive impairment, not otherwise specified | 31 (7.93%) |
| Cognitively normal (volunteers consulting for risk of AD) | 26 (6.65%) |

**Table S4. Correlations of plasma biomarkers with Aβ and tau PET in individuals with two PETs in CADS.**

| **Individuals with Aβ PET(+)** | | | | | |
| --- | --- | --- | --- | --- | --- |
| Blood biomarkers | Aβ PET (Centiloids) | | Tau PET  (Temporal Meta-ROI CTR_z_) | | P value |
|  | ρ | p value | ρ | p value |  |
| Aβ42/40 | 0.030 | 0.786 | 0.147 | 0.177 | 0.446 |
| p-tau217 | 0.263 | 0.014 | 0.697 | <0.001 | **<0.001** |
| p-tau181 | 0.208 | 0.055 | 0.407 | <0.001 | 0.154 |
| p-tau217/Aβ42 | 0.265 | 0.014 | 0.711 | <0.001 | **<0.001** |
| p-tau181/Aβ42 | 0.249 | 0.021 | 0.506 | <0.001 | 0.051 |
| **Individuals with Tau PET(+)** | | | | | |
| Blood biomarkers | Aβ PET (Centiloids) | | Tau PET  (Temporal Meta-ROI CTR_z_) | | P value |
|  | ρ | p value | ρ | p value |  |
| Aβ42/40 | -0.030 | 0.790 | 0.094 | 0.397 | 0.432 |
| p-tau217 | 0.293 | 0.007 | 0.667 | <0.001 | **0.001** |
| p-tau181 | 0.211 | 0.055 | 0.378 | <0.001 | 0.247 |
| p-tau217/Aβ42 | 0.294 | 0.007 | 0.677 | <0.001 | **<0.001** |
| p-tau181/Aβ42 | 0.253 | 0.021 | 0.478 | <0.001 | 0.098 |
